# Observation and density estimation of a large number of skin capillaries using wide-field portable video capillaroscopy and semantic segmentation

**DOI:** 10.1117/1.JBO.28.10.106003

**Published:** 2023-10-24

**Authors:** Baku Takimoto, Kotatsu Bito, Sayaka Hari, Hiroyuki Taguchi, Hideaki Haneishi

**Affiliations:** a Kao Corporation, Analytical Science Research Laboratories, Tokyo, Japan; b Kao Corporation, Digital Business Creation, Corporate Strategy, Tokyo, Japan; c Kao Corporation, Health and Wellness Products Research Laboratories, Tokyo, Japan; d Kao Corporation, R&D Strategy and Planning, Tokyo, Japan; e Chiba University, Center for Frontier Medical Engineering, Chiba, Japan

**Keywords:** skin microcirculation, skin capillary, capillaroscopy, semantic segmentation, U-Net, deep learning

## Abstract

**Significance:**

Skin capillaries are non-invasively observable; their structure and blood flow can reflect tissue and systemic conditions. Quantitative analysis of video-capillaroscopy images yields novel diagnostic methods. Because the capillary structure is heterogeneous, analyzing more capillaries can increase the evaluation reliability.

**Aim:**

We developed a system that can observe and quantify numerous capillaries and verified the performance on human skin.

**Approach:**

We developed a portable video-capillaroscope with a spatial resolution higher than 3.5  μm and a wide field of view (7.4  mm×5.5  mm) and a method to evaluate capillary numbers and areas using U-Net. The model was trained and tested with 22 and 11 cropped images (2.4  mm×1.9  mm) obtained from 11 participants, respectively. They were then applied to the 7.2  mm×5.3  mm images from four participants. Segmentation results were compared to ground-truth at the pixel level and capillary-region level.

**Results:**

Over 1000 capillaries were simultaneously observed using the proposed system. Although pixel-level segmentation performance was low [intersection over union (IoU) = 24.5%], the number and area could be estimated. These values differed among four participants and seven sites, and they changed after skin barrier destruction.

**Conclusions:**

The proposed system allows for observing and quantifying numerous skin capillaries simultaneously, suggesting its potential for evaluating tissue and systemic conditions.

## 1 Introduction

Biological systems have heterogeneous and hierarchical structures that range from microscopic to macroscopic levels, and they possess dynamic properties, such as material transport and deformation. A typical vascular system possesses such spatial and temporal complexity because it has a hierarchical structure that is reflected by microvasculature, including capillaries leading to the aorta and vena cava, in addition to dynamic properties, such as internal blood flow and angiogenesis. As most parts of this system are distributed deep in the body, non-invasive observation is difficult. However, the skin and nailfold capillaries on the body surface can be easily and non-invasively observed under visible light using a microscope (capillaroscope); this ability can be exploited to develop strategies for evaluating the condition of tissues and the entire body.

Thus far, some studies have reported on the relationship between capillary density and structure in capillaroscopy images and diseases. For example, there has been a decrease in the number of skin capillaries in patients with diabetic neuropathy^1^ and their structural abnormalities in patients with diseases, such as basal cell carcinoma and melanoma.^2^ In addition, the skin and nailfold capillaries may reflect the condition of the entire body owing to their continuity to the deep vascular system. For instance, the shapes of the nailfold and labial mucosa capillaries change due to systemic scleroderma^3^^–^^5^ and chronic smoking,^6^ respectively. Recent studies have suggested that COVID-19 may also be detected based on the changes in capillaries.^7^^,^^8^ In capillaroscopy images, only the capillaries containing red blood cells (RBCs) are visible. Accordingly, by regarding observable capillaries as “functioning” capillaries, the quantification of the functional capillary density (FCD) was proposed.^9^ It has been reported that the FCD correlates with the survival rate of patients treated with extracorporeal membrane oxygenation.^10^

However, the complexity and dynamic properties peculiar to biological systems mentioned previously make this application difficult. Because the shape of capillaries and the blood flow within them are heterogeneous for each capillary, observing a large number of capillaries would be useful for a reliable evaluation. Nevertheless, as the capillaries are <10-μm wide, capillaroscopes with a high resolution and, therefore, a limited field of view are generally used, and only a limited number of capillaries can be observed. In typical capillaroscopes, the width of the field of view is <1  mm^6^^,^^11^^–^^15^ and, even in wider cases, it is <3  mm.^5^^,^^7^^,^^16^^–^^19^ By contrast, capillaries branched from one arteriole are distributed in an area of ∼1  mm.^20^ Therefore, observations made with a field of view of <1  mm can be affected by the heterogeneity among arterioles. Considering such heterogeneity, methods such as acquiring images at several sites in the case of the oral cavity^13^ or combining multiple images to create an image with a wide field of view have been reported.^3^^,^^4^^,^^21^ However, these methods involve complicated procedures and high operator and participant loads.

Therefore, if a large number of capillaries can be simultaneously imaged using a wide field of view and their characteristics, such as density, can be quantitatively estimated, a more detailed understanding of the state of the tissue can be obtained. Such a system requires the following elements:

Wide field of view and adequate spatial resolution: In general, there is a trade-off between field of view and resolution. However, it is expected that many capillaries can be simultaneously observed using a wide field of view with an area that is several to several dozen times larger than that of commercial devices and is compatible with the spatial resolution equivalent to or better than that of capillaries with a minimum inner diameter of 3  μm.^20^
Video recording: Because blood flow in capillaries is intermittent and only capillaries containing RBCs are visible, it is desirable to acquire video images rather than still images to characterize capillaries.
Configuration suitable for skin observation: The device used for observing cutaneous capillaries should be configured such that it is less susceptible to the light reflected from the skin surface and maintains stable contact with the skin during observation.
Image processing that extracts capillary regions from videos and calculates parameters: Because many capillaries must be handled, automatic analysis is preferred over manual analysis, which requires time and effort. Various automatic capillary region extraction methods have been proposed, including the use of the minimum values and dispersions at each pixel in multiple frames of video images,^12^^,^^22^ and principal component analysis and the Frangi filter.^17^ However, the structures, such as sweat glands, stains, hair, and dirt, that adhere to the skin co-exist with capillaries in the wide-field images, making it difficult to set optimal image-processing conditions to only detect capillaries. In a video image, RBCs can be seen flowing intermittently, and thus, the capillaries may be extracted as regions with high pixel value variations. However, some capillaries are filled with blood over the entire frame. Hence, all capillaries cannot be extracted based only on the RBC motion. Recent studies have used deep learning for capillary observations, which automatically sets the appropriate parameters for image processing, improving image analysis with fewer human-controlled criteria. Semantic segmentation in image processing is used to identify the object in each pixel of the image.^23^ Recently, vascular region extraction through semantic segmentation has been reported for nailfold capillaries^14^^,^^15^^,^^24^ and retinal vascular networks,^25^ and it is expected to distinguish capillaries from other structures with high accuracy.

Technologies, such as photoacoustic imaging,^26^^–^^28^ optical coherence tomography-angiography (OCT-A),^29^^–^^31^ reflectance confocal microscopy,^32^^,^^33^ and two-photon microscopy,^34^ offer three-dimensional (3D) views of the cutaneous vasculature, and some of them can capture images at resolutions of several millimeters or centimeters.^26^^,^^27^^,^^29^ However, they require expensive and complicated systems that are difficult to operate and may entail long data acquisition times. Therefore, a capillaroscopy system with the aforementioned specifications can potentially be a novel and more versatile diagnostic method.

In this study, we developed a portable device that can observe many skin capillaries with a wide field of view and adequate spatial resolution. Thereafter, we verified that the number and area of more than 1000 skin capillaries can be automatically estimated from the obtained images using semantic segmentation. In this study, U-Net,^35^ which is a well-established semantic segmentation algorithm often used in biology and medicine, was used.

The device comprises a high-resolution camera (4000×3000  pixels) that is capable of recording at a higher resolution than 4K (3840×2160  pixels), a lens with appropriate magnification and numerical aperture, a lens barrel with built-in light sources, and a part for stable contact with the skin. The device generates 30 images at a resolution of 4000×3000  pixels per second, providing a considerable amount of data. Images obtained with this device were subjected to U-Net, and the number and area of capillaries were estimated. After verifying the results based on comparison with those of the manual extraction of capillary regions, the images taken with different participants, sites, and before and after skin barrier destruction as external stimulation were analyzed to evaluate the applicability to the tissue condition assessment.

## 2 Materials and Methods

### 2.1 Construction of a Wide-Field Video-Capillaroscopy System

**Fig. 1:**
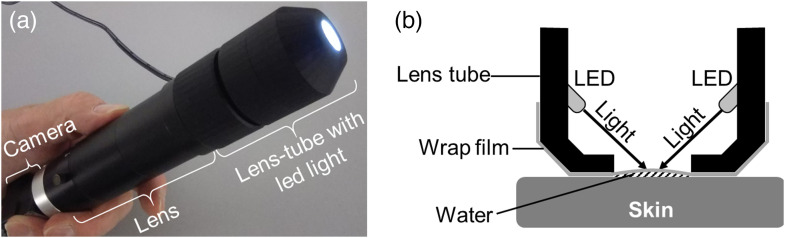
Appearance and configuration of the developed system. (a) Schematic diagram and (b) internal structure of the proposed wide-field portable high-resolution video-capillaroscopy system.

We developed a portable wide-field video-capillaroscopy system capable of imaging skin capillaries over a wider field of view than general capillaroscopy (Fig. 1). By combining a USB 3.0 camera (Toshiba Teli BU1203MC, Tokyo, Japan) having a large number of pixels with a high-resolution telecentric lens (VS Technology x1 VS-TCT1-65/S, Tokyo, Japan, numerical aperture = 0.111), it was possible to image an area of 7.4  mm×5.5  mm at a resolution of 4000×3000  pixels, and the field of view per pixel was 1.85  μm. We evaluated the spatial resolution of the system using a USAF1951 test target (Edmund Optics #58-198, New Jersey). The working distance of the lens was 65 mm, and a cylindrical acrylic lens barrel with a φ12 mm aperture developed using a 3D printer was attached to the tip of the lens so that a focused image could be obtained by simply pressing it against the skin. Four shell-shaped white LEDs (OptoSupply LP-WA4K3131A, Hong Kong, China) were used as light sources inside the lens barrel to be illuminated at an angle of 45 deg to the skin surface. This microscope was connected to a PC with a cable (OMRON SENTECH NU3MBASU3S-2m, Ebina, Japan). The images were displayed and recordings were obtained using a viewer software (TechnoScope MKS-01, Saitama, Japan).

### 2.2 Capturing Video Images of Capillaries

#### 2.2.1 Participants

This study included 11 healthy Japanese men (26 to 55 years old; average age: 40.6 years) who gave their consent through a written form after the experimental procedure was explained to them. Patients with a history of skin diseases, such as atopic dermatitis, contact dermatitis, skin hypersensitivity; those with scratches on the target site that could impede the securing of the measurement site; and those who had participated in other tests with intervention within 1 month before our test were excluded from the study. The left and right inner forearms were used as the measurement sites. The study was conducted following the Helsinki Declaration, and all procedures were approved by the Human Research Ethics Committee of Kao Corporation.

#### 2.2.2 Skin cleaning and barrier destruction

After washing both inner forearms, the excess moisture was removed using paper towels, and then the forearms were acclimatized for 20 min before capturing video images. We evaluated whether the proposed method could capture changes in the blood flow rate before and after skin stimulation. As the stimulus, we employed a barrier destruction treatment through tape-stripping,^36^ which is a mild physical stimulus that has been reported to activate microcirculation post treatment.^37^ Tape-stripping was performed through with cellophane tape (Nichiban Cellotape™, Tokyo, Japan). Transepidermal water loss (TEWL) was used to index the skin barrier function. Before treatment, the TEWL of the skin was measured using a VapoMeter (Delfin Technologies, Kuopio, Finland), and tape-stripping was then performed until the value was doubled (six persons) and tripled (five persons). The seven sites of each participant (four sites on the left and three on the right inner forearm) where these processes were performed were imaged using the system developed in this study. A schematic diagram and photograph of the measurement sites are shown in Figs. S1(a) and S1(b) in the Supplemental Material, respectively.

#### 2.2.3 Capillary imaging

To eliminate obscurity of the capillary structure within the skin due to light reflection from the skin surface, we added a few drops of water to the skin and imaged it through a polyvinylidene chloride wrap film (Kureha NEW Krewrap, Tokyo, Japan). Using this configuration, the refractive index (n) difference between the stratum corneum (n=1.55) and surrounding medium (air: n=1.0, water: n=1.33) decreased; thus, the light reflection at the skin surface was suppressed, and the visibility of the capillaries inside the skin increased. In addition, the reduction in the capillary blood flow owing to pressure inside the field of view could be avoided using a soft wrap film instead of a hard window material, such as glass, and contacting the outer part of the field of view against the skin.

Considering the changes in the presence or absence of RBCs in capillaries over time, we acquired video images at a frame rate of 30 frames per second for 2 min. Thereafter, we used 5 s (150 frames) with little influence from body movement in the early stage of the video images for image processing. Subsequently, we analyzed the images captured before and after skin barrier destruction. Still images captured without the water and wrap film are shown in Fig. S2 in the Supplemental Material.

### 2.3 Image Processing

**Fig. 2:**
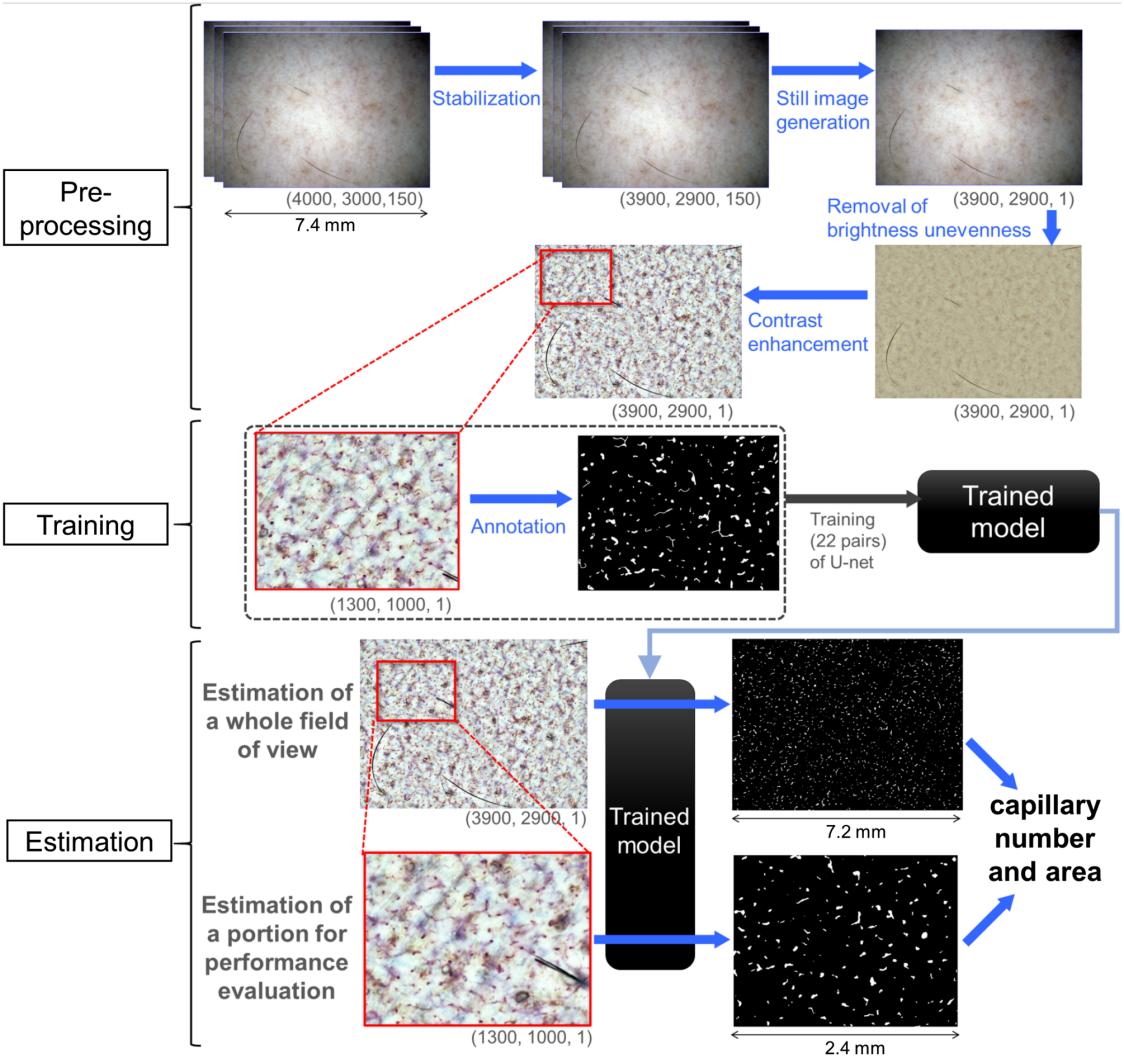
Schematic diagram of image processing. Pre-processing, U-Net training, and capillary area detection using the trained model are shown. Image size (horizontal pixel number, vertical pixel number, frames) is shown in the lower right area of figure.

We performed image processing to extract capillaries from the video images in two stages: pretreatment of images (image stabilization, generation of a still image with capillary structure from the video image, removal of brightness unevenness, and contrast enhancement) and detection of capillary regions using semantic segmentation and quantifying their area and number. The image-processing procedure is shown in Fig. 2, and a detailed explanation is given below. We used programs written in Python for image processing in the following execution environment: Windows 10 Pro 64-bit operating system with a Core i7-6950X 3.00/3.50 GHz processor and 128 GB of memory.

### 2.4 Image Stabilization

In the video images captured, we observed a motion blur resulting from the body movement of either the operator or the participant. To correct this blur and stabilize the image, we used template matching. The central 3900×2900  pixels of the first frame of the video image were extracted as the template. In 150 frames of the video image of 4000×3000  pixels, the areas of size 3900×2900  pixels with high similarity to the template were extracted. By creating a video image using the extracted images, we obtained the stabilized video image without motion blur. We used the matchTemplate function of OpenCV (version 4.1.0)^38^ for processing and the TM_CCOEFF_NORMED method to calculate the similarity.

### 2.5 Generation of Still Images from Video Images

In the video images of the skin capillaries, the capillaries themselves cannot be distinguished from their surroundings and can only be recognized when RBCs pass through them. Therefore, if a single frame is extracted, the capillary structure may have gaps at locations with no RBCs. Therefore, we processed 150 frames of images to depict capillary regions without gaps as places through which RBCs have passed. A method of averaging the 150 frames could be considered. However, if RBCs passed only rarely, the contrast would be low and the vascular regions might not be sufficiently depicted. Considering this, we focused on the minimum instead of average pixel values and processed the image as follows. In the color image, RBCs appeared redder than the surroundings. This makes the pixel value of the green channel at the RBCs smaller than that at the periphery when the image is divided into its constituent red (R), green (G), and blue (B) channels. By focusing on a single pixel and recording the color of the frame wherein the pixel value of the G channel is the lowest across the 150 frames, the information from the frame wherein RBCs exist can be obtained. Thus, a still image in the area where RBCs flowed in 150 frames was generated.

### 2.6 Removal of Brightness Unevenness

In the images captured during this study, the peripheral parts were darker than the central parts due to the unevenness of the skin surface shape and the irradiated light intensity. In our system, this unevenness of brightness was conspicuous due to the wide field of view compared with the conventional system. This unevenness of brightness might compromise capillary visibility and make manual annotation for deep learning inaccurate. Therefore, we removed the brightness unevenness as follows:

(i) A still image was created using the aforementioned process and divided into its R, G, and B channels.
(ii) A mean filter with a kernel size (radius) of 300 pixels was applied to extract the uneven brightness.
(iii) Images with even brightness were obtained by dividing each of the R, G, and B channels of the original image by the corresponding images after mean filtering.
(iv) The R, G, and B channel images were merged to generate a color image.

### 2.7 Contrast Enhancement

Drops and mistakes are likely to occur during annotation if the vascular area is unclear. Therefore, contrast enhancement was performed on still images after correcting the brightness to facilitate annotation. This process was performed through histogram equalization (using OpenCV’s equalizeHist function) and gamma correction (γ=0.3) for each of the R, G, and B channels.

### 2.8 Semantic Segmentation of Capillary Regions

In this study, the U-Net, which is an existing segmentation algorithm, was used to extract the capillary regions. The U-Net has a U-shaped structure and can even accurately classify fine structures by maintaining the position information of each pixel during convolution and deconvolution. In this method, the input and ground-truth images containing the object’s annotated position are used as pairs to generate the trained model for image processing. Thereafter, the trained model is used on images different from those used for training, and the target objects are automatically identified and detected. This method was executed on Python (version 3.6.7). NumPy (version 1.16.2), Keras (version 2.1.6), and TensorFlow (version 1.10.0) were used as libraries to run U-Net.

#### 2.8.1 Annotation

We manually painted the capillary regions in the still images white (pixel value 255) and the remaining regions black (pixel value 0) using a liquid crystal pen tablet (Wacom Cintiq 16 DTK1660K0D, Kazo, Japan) and image-processing software (Adobe Photoshop, California) for annotation. Annotation was performed by a single annotator who was skilled in imaging cutaneous capillaries. In the images after contrast enhancement, structures with extreme redness compared with the surroundings and relatively clear outlines were annotated as capillaries. Because the load was too large for the 3900×2900  pixel images to be used, areas of size 1300×1000  pixels (equivalent to 2.4  mm×1.9  mm) in the images were extracted and used. For training, we used 22 images of the 11 participants before and after tape-stripping. Furthermore, we used 11 images either before or after tape-stripping of each participant in the test process to evaluate the estimation accuracy. The sites of the training and test images and whether they were captured before or after tape-stripping are shown in Fig. S1(c) in the Supplemental Material.

#### 2.8.2 Training and estimation of skin capillary regions

In general, in the training process of deep learning, numerous images (patch images) are created from the input images to increase the number of images. This study used 150 images that were created by extracting areas of size 512×512  pixels at random positions from 22 images of 1300×1000  pixels. The batch size and number of epochs were 16 and 50, respectively. The loss function used during training is shown in Fig. S3 in the Supplemental Material.

We then performed semantic segmentation with the trained model for 11 test images. In this process, the segmentation was performed for the upper left 512×512  pixels of the 1300×1000  pixel image and then sequentially for areas that were moved 50 pixels to the right. After processing was performed on the upper right side of the image, the process was performed by moving 50 pixels in the right direction in the same manner from the place where the 50 pixels were moved downward from the initial position. By repeating this process sequentially, we estimated every pixel of the 512×512-pixel image. In each of the extracted 512×512  pixels, the areas other than the capillaries were drawn in black, and the capillary regions were white. Once the processing was completed for the entire image, the estimated images were merged to maintain the original position and create a single image of 1300×1000  pixels. Subsequently, the image was binarized to create an image wherein the areas other than the capillaries were drawn in black and the capillary areas in white. Further, to confirm that the capillary region could be extracted over a wide field of view, a similar estimation process was performed using an image of 3900×2900  pixels (7.2  mm×5.3  mm) as input.

#### 2.8.3 Evaluation of estimation performance

We evaluated the performance of capillary region estimation through U-Net by comparing the quantitative capillary variables for the 11 test images with manually annotated ground-truth images. The number and area (total and average) of the extracted capillary regions were calculated as the capillary variables through blob analysis using the connectedComponentsWithStats function of OpenCV and were compared.

In addition, after determining the true positives (TP), true negatives (TN), false positives (FP), and false negatives (FN) for each pixel, the accuracy, precision, recall, specificity, Sørensen–Dice index (Dice), and intersection over union (IoU) were calculated to evaluate the segmentation performance at the pixel level.

Even when capillaries identical to those in ground-truth could be detected by U-Net, some persisting concerns were that the regions would not match completely due to the unclear outlines of the capillaries in the image and their small widths, resulting in a significant decrease in the pixel-level accuracy index mentioned previously. Therefore, in addition to the pixel-level evaluation, the capillary region-level, extraction performance was also evaluated. Among the capillary regions in ground-truth, regions in which even some pixels were detected by U-Net were counted as TP regions, and those in which no pixels were detected by U-Net were counted as FN regions. Regions that were detected as capillary regions in U-Net but were not capillaries in ground-truth were counted as FP regions. We then used numbers of these regions to calculate precision, recall, and Dice. However, TN and IoU, an index based on TN, were not used due to definitional difficulties. A detailed explanation of this analysis is given in Figs. S4 and S5 in the Supplemental Material.

### 2.9 Evaluation of Differences Between Participants and Sites and Changes due to Skin Barrier Destruction

To analyze the differences in capillary variables among individuals and sites, we evaluated the variables at seven locations on the inner forearm. Further, to evaluate the external stimulation before and after skin barrier destruction through tape-stripping of each participant, we used data of four of the five participants who underwent barrier destruction through tape-stripping until TEWL was tripled, excluding one who exhibited significant blurring in the video image. The differences were evaluated using a paired t-test.

## 3 Results and Discussion

### 3.1 Wide-Field Imaging of Skin Capillaries

**Fig. 3:**
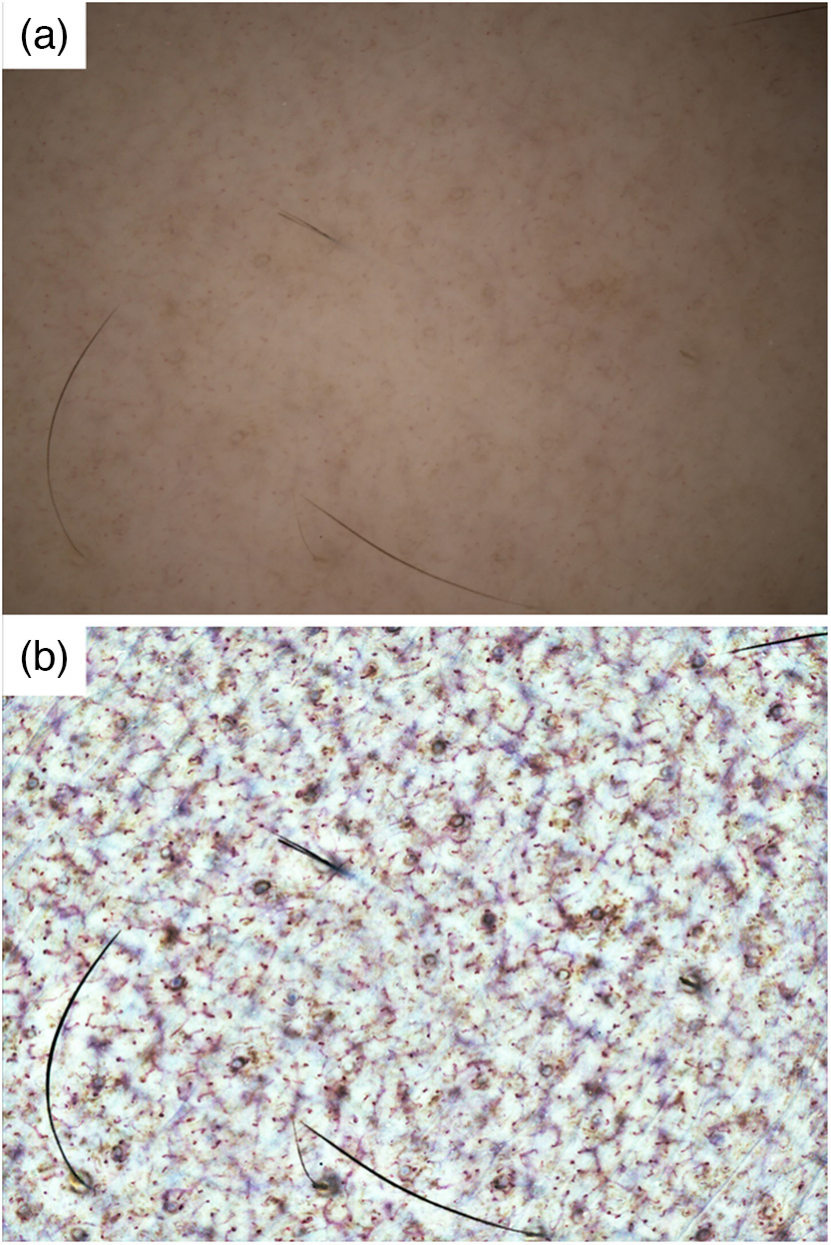
Large area skin capillary imaging result. Image of whole field of view of 7.2  mm×5.3  mm (a) before and (b) after removal of brightness unevenness and contrast enhancement.

By evaluating the spatial resolution of the wide-field video capillaroscopy using the USAF test target, a gap of 3.5  μm could be observed. Although this resolution is not higher than that of some commercial capillaroscopy equipment, such as Dino-Lite (IDCP Medtech, Almere, Thenetherland), it is comparable to the capillary width, with inner and outer diameters that are 3 and 10  μm,^20^ respectively. These values are sufficient for capillary observation. Observations of the skin before and after tape-stripping via water and a wrap film indicated that the skin texture and the specular highlights were suppressed throughout the entire field of view (Fig. 3). In addition, after contrast enhancement, many capillaries were observed in addition to stains and sweat glands. This result shows that, using the proposed system, the capillary structures could be observed in a wide field of view of 7.4  mm×5.5  mm. Owing to its wide field of view, it was possible to virtually observe the same field of view repeatedly before and after skin barrier destruction without using complicated operations, such as switching to a low-magnification configuration. The sample images captured with and without water and wrap film are shown in Fig. S2 in the Supplemental Material.

**Fig. 4:**
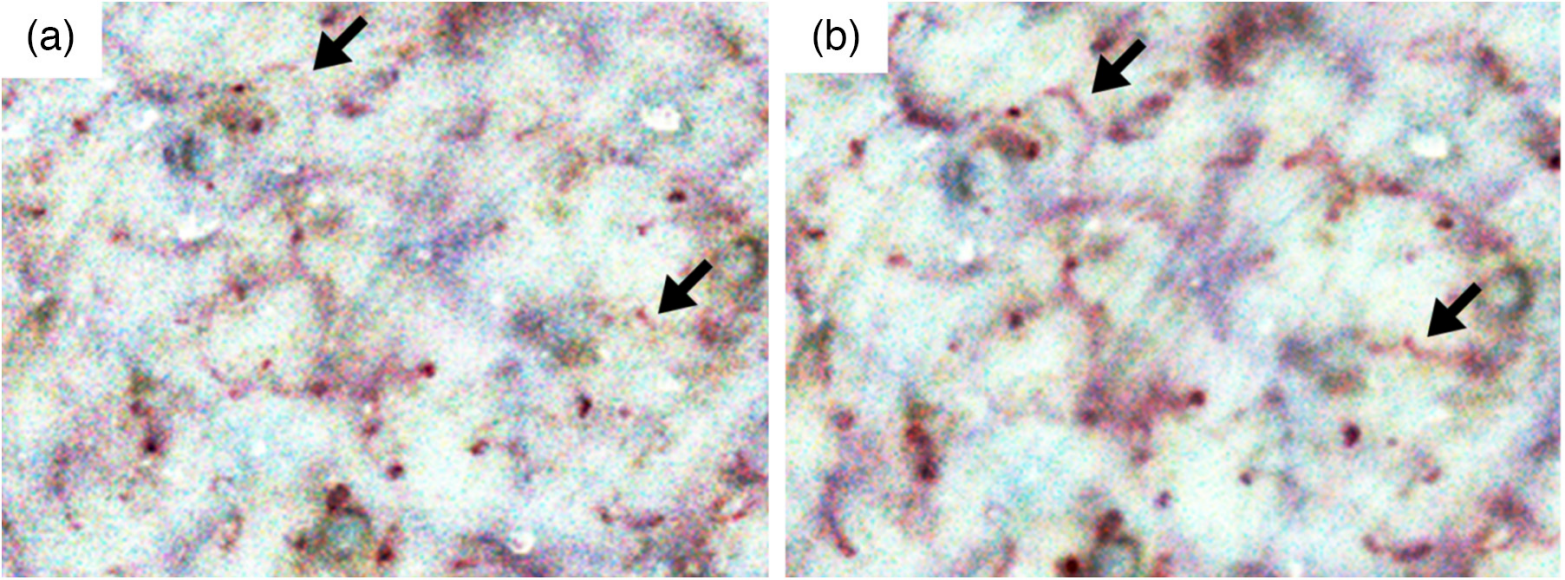
Visibility of capillaries in a still image generated using 150 frames of a video image. Examples of contrast-enhanced images of (a) a single-frame and (b) a still image prepared using 150 frames of a video image taken after barrier destruction with water and wrap film. Enlarged images of size 800×600  pixels (3.7  mm×2.8  mm) at the same site are shown. Visibility of the capillaries shown with arrows in (b) is better than that in (a).

In the single-frame image, RBCs were partially present in the capillaries in some areas, and the capillaries were observed indistinctly. However, even at such a site, we continuously monitored the capillary structure in the still image generated from 150 frames (Fig. 4). If the density of the observed capillaries can be quantified through image analysis, the variables corresponding to the FCD can be extracted from a wider field of view than that used in existing technology.

Although it was not the focus of this study, because RBCs were visible in some frames and not in others for the same capillaries, dynamic indicators such as blood flow velocity and supply rate to the capillaries could be determined.

### 3.2 Estimation Results for the Capillary Region

**Fig. 5:**
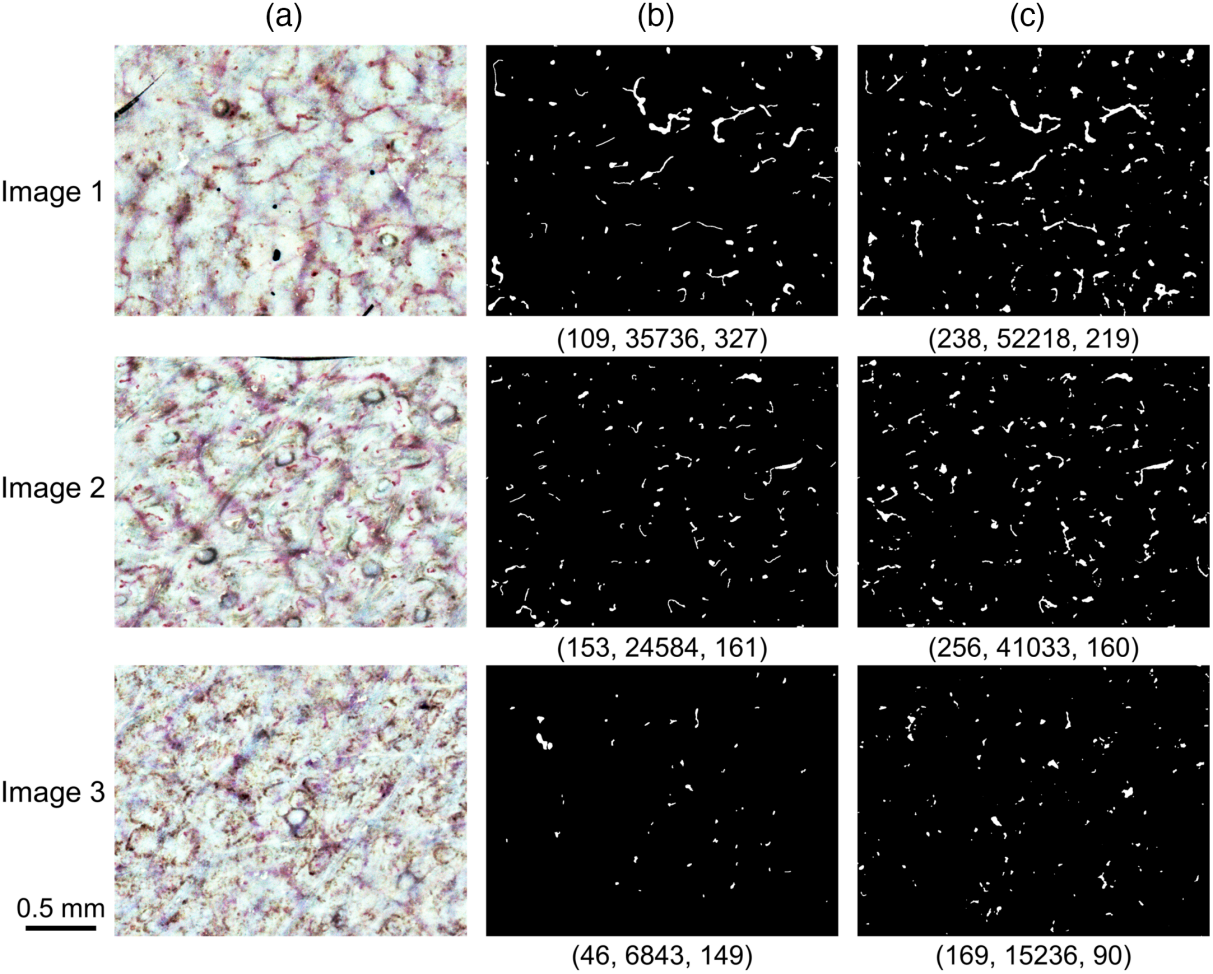
Semantic segmentation of capillaries. (a) Input images generated using 150 frames and enhanced contrast of different participants and sites. (b) Ground-truth images prepared manually from each input image. (c) Detection results after segmentation. Three examples of 11 results are shown. Number of capillary regions, total area (pixel), and average area (pixel), respectively, are mentioned under each binarized image.

An example of the detected capillary regions after semantic segmentation is shown with the input and ground-truth images in Fig. 5. Some capillary regions detected by U-Net appeared to correspond to those in the ground-truth at first glance while other areas did not. Therefore, for a quantitative analysis of the correspondence between detection results and ground-truth, we evaluated the degree of agreement at the pixel level and the capillary region level, the correlation between the number and area of capillaries obtained in both, and the Bland–Altman plot.

### 3.3 Verification of Estimation Performance at the Pixel Level

The estimation performance at the pixel level was calculated using indices that are generally used for segmentation performance evaluation. Here, we used images of 1300×1000  pixels (equivalent to 2.4  mm×1.9  mm) that were extracted from the full field of view. The accuracy, precision, recall, specificity, Dice, and IoU values were calculated for each image, along with the average values between images. Although accuracy and specificity were high (96.9% and 98.0%, respectively), precision, recall, Dice, and IoU were low (30.2, 47.1, 35.7, and 24.5, respectively). In many capillaroscopic images, the contours of skin capillaries were unclear. Therefore, both the U-Net and manual annotation generated errors in the width of the extracted blood vessels. Unlike the case wherein a car is detected through an in-vehicle camera, the capillary was approximately a few pixels thick in the image used in this study. Therefore, although the error was only a few pixels, it could contribute significantly to TP, resulting in low precision, recall, Dice, and IoU values. However, even after obtaining such errors, if the same capillaries as those in the manual analysis are extracted, it should be possible to estimate the same number and area of capillaries as those estimated through the manual analysis. Thus, the proposed method may be useful for characterizing capillaries, such as FCDs. This attribute is verified in the next section.

### 3.4 Verification of Estimation Performance at the Capillary Region Level

As noted previously, when the overlap of regions between ground-truth and U-Net estimation was not perfect, the traditional measures (e.g., Dice and IoU) at a pixel level were significantly reduced due to the small widths of the capillary structures in our images. In such cases, the same capillaries could have been detected.

To verify whether we could extract capillary regions overlapping the ground-truth even partially, we calculated TP, FP, and FN for each capillary and calculated precision, recall, and Dice (Figs. S4 and S5 in the Supplemental Material). As a result, precision, recall, and Dice were 44.2%, 94.6%, and 58.0%, respectively, and all values improved compared with the pixel-level evaluation (30.2, 47.1, and 35.7). In particular, the high value of recall indicated that most manually annotated capillary regions could be detected. On the other hand, precision and Dice showed only slight improvements from the pixel-level values, indicating that there were many areas of FP. A further improvement in performance is expected by expanding the number of input images and optimizing parameters to improve the trained model and reduce FP.

### 3.5 Verification of Capillary Number and Area Estimation Performance

**Fig. 6:**
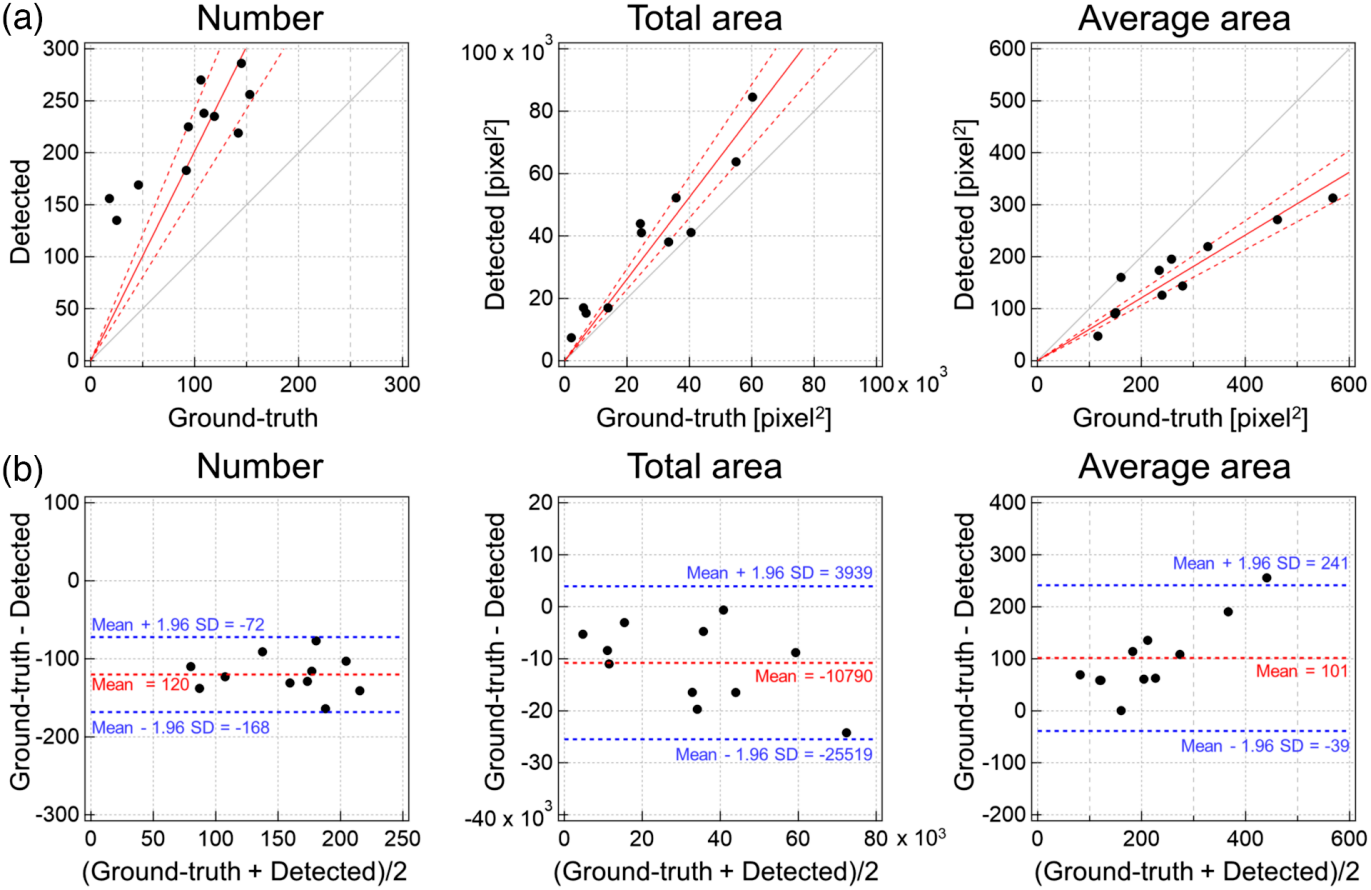
Comparison of capillary area detection results obtained through U-Net and ground-truth obtained through manual annotation: (a) number, total area, and average area of regions detected using U-Net plotted to ground-truth. Red solid, red dashed, and gray solid lines represent the regression line, 95% confidence interval, and y=x line, respectively. (b) Bland–Altman plot of each parameter based on ground-truth and the values detected by U-Net. Red and blue dashed lines represent the mean difference between the ground-truth and detection result, and ±1.96 standard deviations of differences that indicate the LoA.

Using the same images as those in the previous section, the number and area (total and average) of capillary regions estimated by U-Net were compared with the manual analysis results. The number and area of extracted capillary regions correlated well with those of manually annotated regions [Fig. 6(a)]. We obtained the regression equations for the number, total area, and average area as y=2.014x (R=0.96), y=1.311x (R=0.99), and y=0.604x (R=0.99), respectively (y and x are values of regions detected using U-Net and those annotated manually, respectively). In Fig. 6(a), the discrepancy between the regression line and y=x was larger for the total area than that for the number. We assumed that this may be attributed to the difficulty in determining whether it was a single or different capillary when the linear capillaries were partially blurred in the image shown in Fig. 4(b). Even in such cases, the area estimation accuracy was considered to be higher because the same capillaries could be extracted using both methods. We assumed that the regression equation of the total area estimation did not agree with y=x owing to estimation errors of width because of the blurred capillary outlines.

The Bland–Altman plot^39^ calculates the agreement interval, which was within 95% of the differences of the AI, compared to the ground-truth, fall [Fig. 6(b)]. In the plot for the number of capillaries in this study, the mean difference between the ground-truth and detection results was 120, and all points were within the 95% limit of agreement (LoA) (−168 to −72). The total area was also distributed with the mean difference of −10,790 and with all points within the 95% LoA (−25,519 to 3939). For the mean area, the mean difference was 101, and all but one of the data points were within the LoA (−39 to 241). These results indicate that the estimations of the number and area were similar to manual annotations performed by a human.

**Fig. 7:**
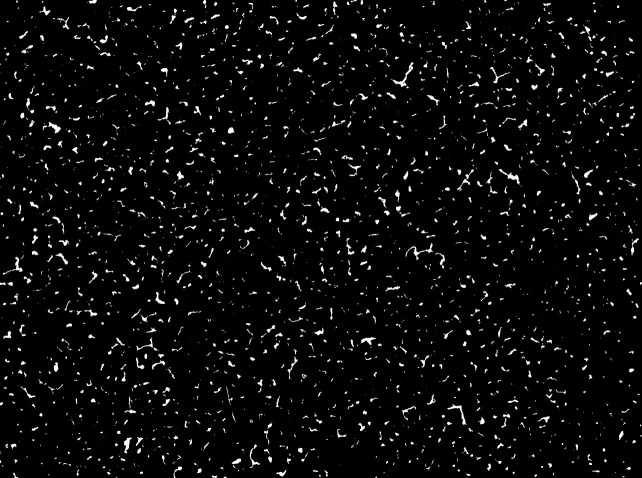
Semantic segmentation result of the large-area image. Segmentation result for the image shown in Fig. 3(a) with a whole field of view (7.2  mm×5.3  mm, 3900×2900  pixels).

Subsequently, we verified whether the capillary regions could be similarly extracted with a wide field of view of 3900×2900  pixels (equivalent to 7.2  mm×5.3  mm). Consequently, capillary regions were extracted from the entire image (Fig. 7). Because the regression line in Fig. 6 did not agree with y=x, instead of taking the detection results in U-Net as the number and density of capillaries as is, we substituted the detection results into y in the above-mentioned regression equations to obtain values corresponding to x.

As a result, in the case of Fig. 7, we estimated the number, total area, and average area as 1234, 428, 279, and 379 pixels, respectively, in the 7.2  mm×5.3  mm (4000×3000  pixels) region. This result shows that more than 1000 capillaries can be quantified from a wide field of view using the proposed method. During microscopic observation of capillaries under visible light, they appear blurry even if the depth increases slightly due to light scattering on the skin. Due to the limited depth of field of the imaging system as well as the aforementioned factor, capillaries distributed at a uniform depth near the very surface layer were observed.

### 3.6 Evaluation of Differences Between Locations and Participants

**Fig. 8:**
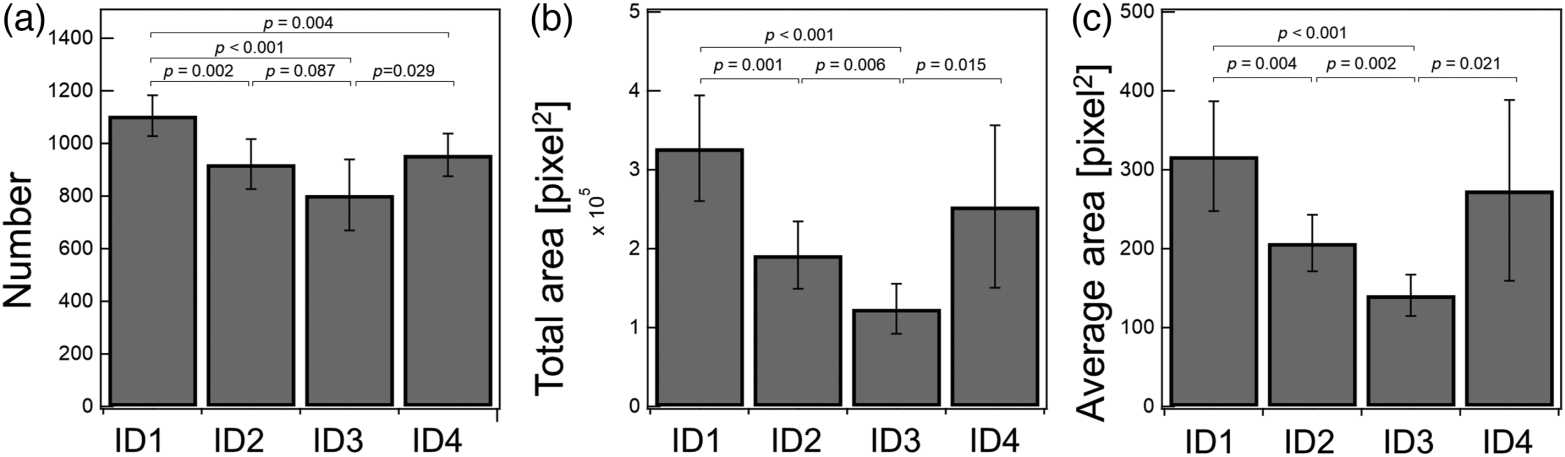
Number and area of capillaries predicted before barrier destruction. (a) Estimated number, (b) total area, and (c) averaged area for four participants who damaged the stratum corneum by tape-stripping until TEWL was three times the initial value. Average values of seven places are shown, and the error bar is the standard deviation. The p values less than 0.1 are shown.

The variables of the capillaries at seven locations in the inner forearm of each participant under normal conditions were compared (Fig. 8). For each participant, ∼1000 capillaries were estimated. There was variation in each location, even for the same participant. It is known that the laser Doppler flowmetry values that measure the blood flow, including the vessels (arterioles, venules, etc.) deeper than the capillary, vary considerably for each measurement point.^40^ It is also known that laser Doppler imaging in a 2.5-cm square ($6.25\;{\mathrm{cm}}^{2}$) region exhibits uneven blood flow.^41^ The capillaries targeted in this study may also exhibit heterogeneity on a similar scale, and such a difference may reflect the local condition of the tissue at each location (Fig. S1 in the Supplemental Material).

Although the proposed method can image a wide field of view of 7.4  mm×5.5  mm wide, considering the difference between locations, it is desirable to take multiple locations to characterize the individual participants. In participants 2 and 3, the total capillary and average areas were small compared with participant 1. This difference may be used to characterize the skin condition and microcirculatory function of each participant.

### 3.7 Changes in Observed Capillaries due to Barrier Destruction

**Fig. 9:**
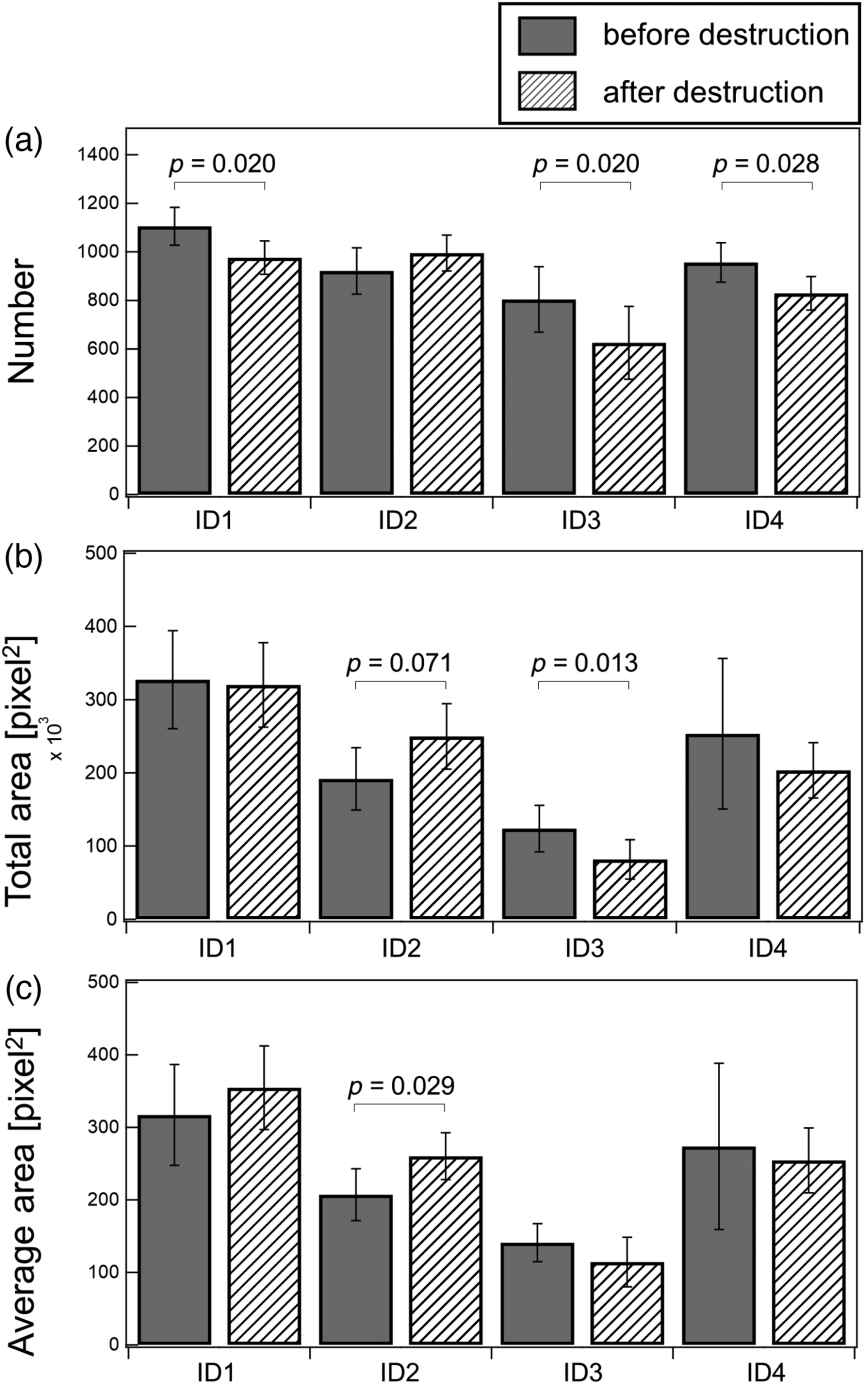
Change in predicted capillaries after skin barrier destruction. (a) Estimated number, (b) total area, and (c) average area before and after skin barrier destruction in four participants, similar to Fig. 8. Average values of seven places are shown, and error bar indicates the standard deviation. p values less than 0.1 are shown.

For each participant, the number and area (total and average) of observed capillaries before and immediately after barrier destruction by means of tape-stripping at the exact location were calculated (Fig. 9). As a result, the number of observed capillaries decreased in participants 1, 3, and 4, whereas in participant 2, it increased. The capillaries’ visibility depends on their RBC content. Thus, this result suggests that the effect of barrier destruction on capillary blood flow varies among individuals. Such differences may help characterize the responsiveness of an individual’s skin microcirculation.

### 3.8 Advantages and Future Challenges of the Proposed Method

We proposed a method for observing and quantifying many capillaries simultaneously with a wide field of view. This method may provide more reliable capillary indices than conventional capillaroscopy. Other modalities used for skin capillary observation include photoacoustic imaging, OCT-A, reflectance confocal microscopy, and two-photon microscopy. These systems require specialized and expensive components, such as complicated and precisely controlled optics, lasers, and ultrasonic transducers. The proposed system comprises simpler and cheaper elements and has a greater potential for clinical applications. In addition, because the shape and distribution of individual capillaries can be directly visualized, it can provide information that cannot be obtained through laser Doppler flowmetry, which computes average blood flow values over a large number of vessels.

This study proposed a technique that can lead to the development of a future diagnostic technique with such advantages, and there is considerable room for future verification. One such consideration is the validation of the capillaries observed and quantified using the proposed method based on a comparison with existing methods. For example, Kelly et al. used a capillaroscopy with a field of view width of ∼1.5  mm and reported that the capillary density was 42 to 50 capillaries per ${\mathrm{mm}}^{2}$ in the normal skin of the inner forearm.^16^ This corresponds to ∼1600 to 1900 capillaries in the field of view of our estimation. Gronenschild et al. also reported 30 to 100 capillaries per ${\mathrm{mm}}^{2}$ within observations of fingers with a field of view of less than 3 mm, which corresponds to ∼1100 to 3800 capillaries in our system.^12^ Although our results (Fig. 6) showed variability among participants, the estimated numbers were probably smaller than the ones reported owing to differences in the body part or participant characteristics, along with the dependency of the detected capillaries on the device specification. Therefore, the same sites as those observed by existing devices must be observed, and the capillaries that can be quantified must be identified. Although the proposed device has a resolution (<3.5  μm) that allows for discriminating the outer diameter of capillaries (>10  μm), it is difficult to discriminate it if two or more are adjacent to each other or if they overlap. As this discrimination can potentially cause errors in capillary count, observing with another device may be useful for verifying and improving the results. In addition to a higher magnification capillaroscopy, photoacoustic imaging, OCT-A, reflectance confocal microscopy, and two-photon microscopy are useful for obtaining the ground-truth of the 3D capillary structure that cannot be observed using the proposed system.

In addition, in this study, skin was observed after tape-stripping to confirm the response to physical stimuli, but contrary to expectations, a decrease in the number and area of capillaries was observed. Based on reports in the literature^37^ wherein it is stated that tape stripping is followed by a mild inflammatory response and an immediate increase in blood flow in the microvasculature, an increase in the number and area of capillaries after stripping was expected. However, in the results of this study, the opposite was observed in some cases. Because there is limited information on the response of blood flow after tape stripping, it is desirable to understand the phenomenon by comparing it with new findings in the future and using the proposed method in combination with other blood flow measurement methods.

Furthermore, the number and characteristics of the participants included in this study were limited. Their skin types^42^ were not assessed precisely but were roughly in the range of type 2 to 4. Expanding the data to include different skin types, the presence of skin diseases, and various body parts and measurement sites (Fig. S1) is expected to improve accuracy and robustness.

## 4 Conclusion

In this study, we developed a wide-field portable video-capillaroscope that can image more than 1000 capillaries with a field of view several tens of times that of a general-purpose capillaroscope. We proposed a method for automatically extracting the capillary variables from the obtained images using semantic segmentation based on deep learning. The density (number and area) of the capillaries estimated by this method correlated with the manual analysis results; in particular, the total area had a high correlation.

The application of the proposed method to each of the seven sites of the four participants gave different capillary feature values between the participants and sites. Thus, it may be used to evaluate microcirculation functions between individuals and sites. In addition, there was a change owing to skin barrier destruction by tape-stripping, and there were individual differences in the degree of this change. Evaluating the response to such perturbations may better assess the effects, such as drugs and treatments.

In addition, because the proposed method can analyze a wide field of view at once, it is expected to possess the following advantages over existing methods: (1) as many capillaries can be imaged and analyzed at once, there is a possibility that heterogeneous capillary parameters can be calculated more reliably; (2) as capillaries branched from multiple arterioles and venules can be analyzed at once, it may be possible to evaluate the variability in the function of each arteriole and venule; and (3) as the proposed method takes a wide field of view, it may be easy to reproduce the field of view, which is difficult with general-purpose equipment.

## Code, Data, and Materials Availability

The code and data are available upon reasonable request.
