# Supplementary material for: Observation and density estimation of a large number of skin capillaries using wide-field portable video capillaroscopy and semantic segmentation
Source: J Biomed Opt. 2023 Oct 24;28(10):106003. doi: 10.1117/1.JBO.28.10.106003 (PMC10594543; doi:10.1117/1.JBO.28.10.106003)

Supplementary material

Figure S1. Measurement sites. (a) Schematic of the measurement sites within the ’ inner forearm of the participant, (b) Photograph of their arm. The black dots are used as landmarks, and the images were captured using the proposed device within the area. The squares surrounded by dots correspond to sites B, C, and D from top to bottom. (c) Participants, sites, and conditions (before or after tape-stripping) from which images were caputers for U-net training and testing.

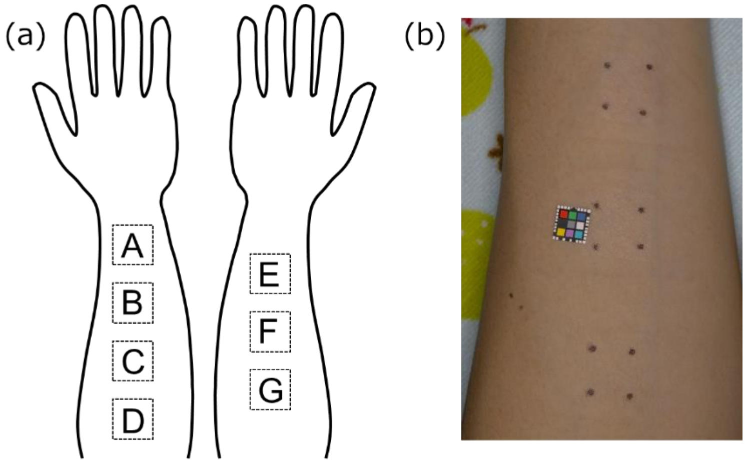

(c)

	training images				test images	
participant #	site	condition	site	condition	site	condition
001	C	before	G	after	B	after
002	G	before	B	after	D	after
003	C	before	B	after	E	after
004	A	before	C	after	F	before
005	A	before	B	after	E	before
006	E	before	B	after	D	before
007	A	before	E	after	B	after
008	A	before	B	after	C	after
009	A	before	A	after	C	after
010	A	before	A	after	D	before
011	B	before	E	after	D	after

Figure S2. Skin observation examples obtained using the developed system with and without water and wrap film, and before and after barrier destruction. The skin surface was observed using a developed system without water and wrap film before barrier destruction with a field of view of 7.4 mm × 5.5 mm (a). The digital zoom (1 mm square region shown with a solid yellow line) of this image showed the specular highlights of the uneven structure of the skin (b). After barrier destruction, the texture became less noticeable in the entire field of view (c) and in the enlarged image (d), and the reflection due to the uneven structure decreased while scales were observed. Thus, although minute morphological changes on the skin surface could be observed with a wide field of view, the visibility of the capillaries inside the skin was lower than that of the reflection and scales and was not suitable for analyzing the vascular structure. In the images obtained with water and wrap film before (e) and after (h) barrier destruction, the surface structure became invisible. In the respective enlarged (f, i) and contrast-enhanced (g, j) images, inner structures such as capillary are seen clearly. These images are single-frame images from the captured video images. It should be noted that the enlarged images (b, d, f, g, i, j) show the same site, with the benefit of the large field of view of the system.

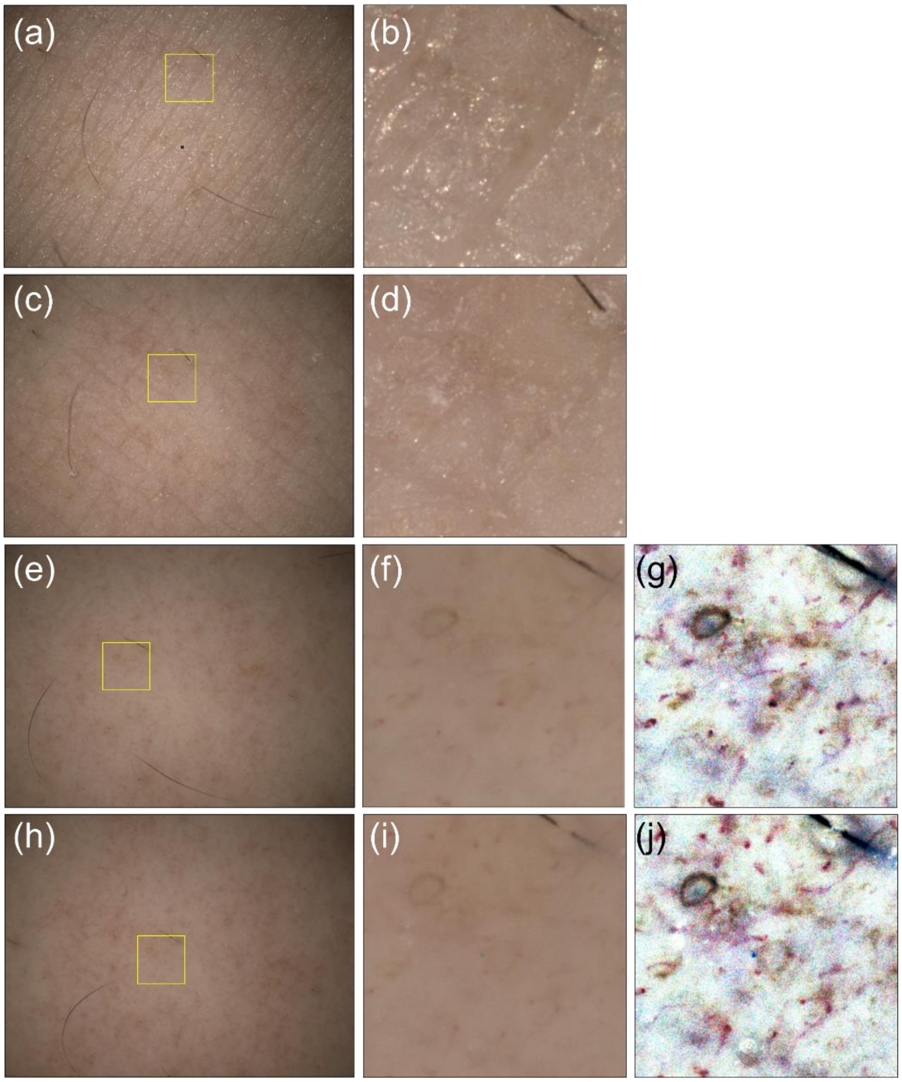
(a)
(b)
(c)
(d)
(e)
(f)
(g)
(h)
(i)
(j)

Figure S3. Loss function plot. Change in the loss function with respect to the number of epochs during U-Net training.

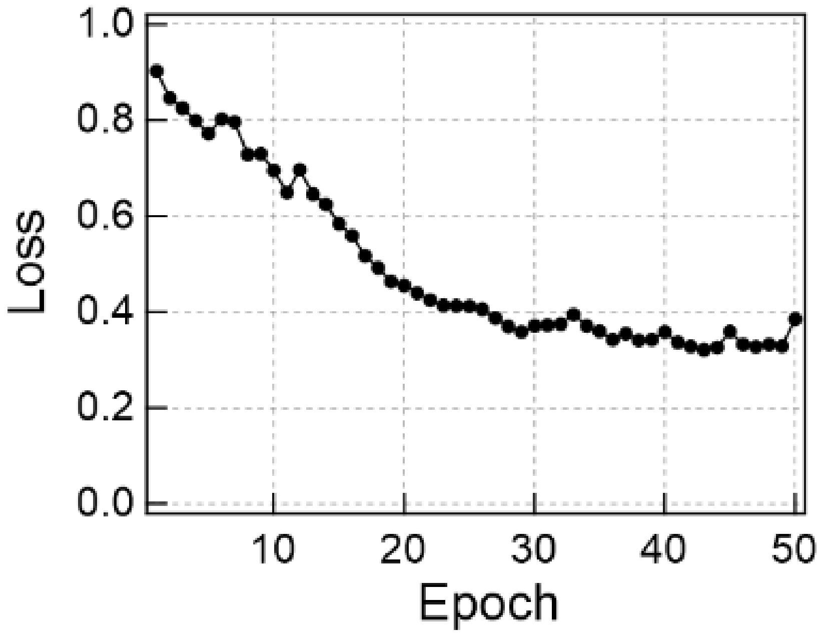

Figure S4. Evaluation method of capillary region detection. In §3-3, it may be questionable whether U-net and manual annotation could be extracted with the same quality, given the low performance metric values such as pixel-level IoU. Therefore, in addition to the pixel-level, we evaluated whether we could detect the corresponding regions for each capillary, as described in §2-8-3 and §3-4. The following figure shows the overlap of detected capillaries by U-Net and the capillary regions in the ground-truth images of 1300 × 1000 pixels (2.4 mm × 1.9 mm). Areas where both overlapped are shown in yellow, areas seen only in U-Net output are shown in red, and areas seen only in ground-truth are shown in green. Continuous regions containing yellow and green (capillary regions in ground-truth that even partially contain the region detected by U-Net) were defined as TP regions. The regions consisting only of red were counted as FP regions and those consisting only of green were counted as FN regions. Based on these, recall, precision, and Dice were calculated as shown in Table S5.

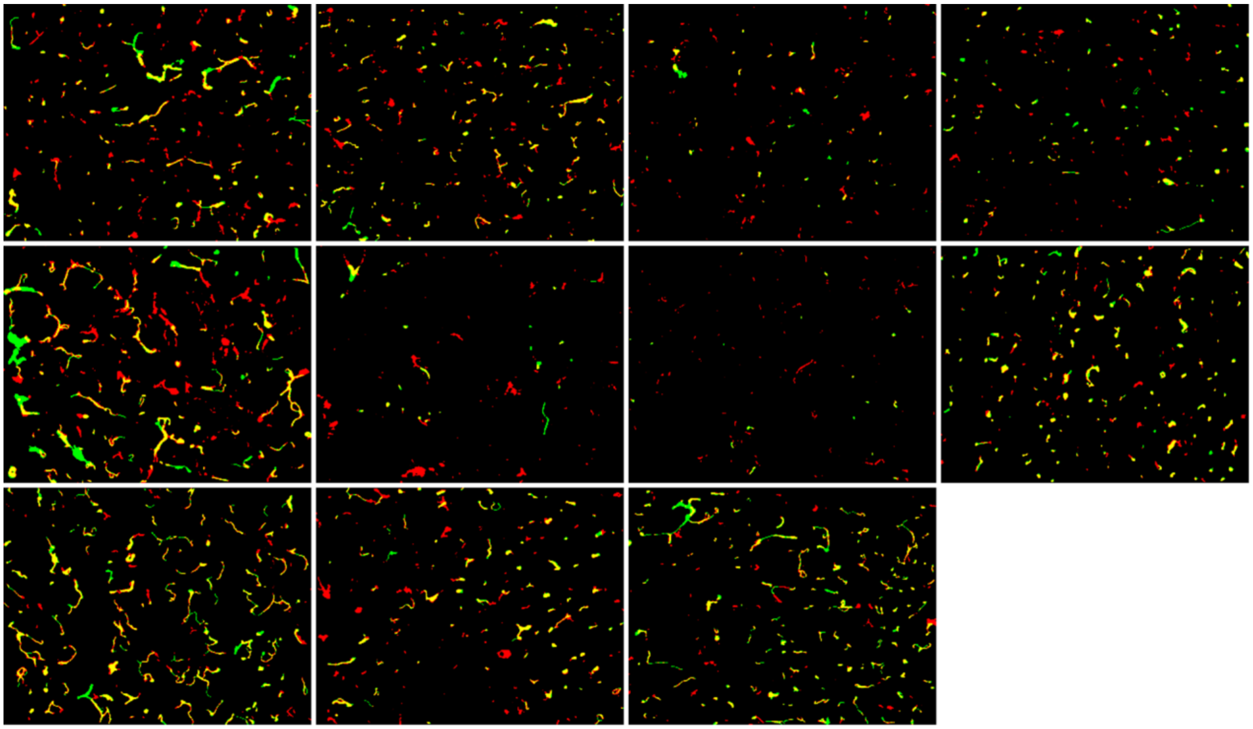

Table S5. Evaluation result of capillary region detection.

For the test images obtained from each subject shown in S1(c), the numbers of TP, FN, and FP regions were calculated at capillary-region level using the method described in S4. The results and recall, precision, and Dice calculated from these numbers are shown in the table.

participant #	TP	FN	FP	recall	precision	Dice
001	105	4	126	0.963	0.455	0.618
002	147	6	99	0.961	0.598	0.737
003	40	6	128	0.870	0.238	0.374
004	84	8	96	0.913	0.467	0.618
005	103	3	143	0.972	0.419	0.585
006	21	4	116	0.840	0.153	0.259
007	17	1	138	0.944	0.110	0.197
008	136	6	72	0.958	0.654	0.777
009	118	1	60	0.992	0.663	0.795
010	94	0	115	1.000	0.450	0.620
011	145	0	74	1.000	0.662	0.797
average				0.947	0.442	0.580
standard deviation				0.052	0.201	0.213

Figure S6. Extra images. Sample images of inputs and capillary detection result extraction for cases with large and small capillary numbers and area estimations. The images show the entire field of view after image stabilization and contrast enhancement. (a) Participant 1 after tape-stripping with 1000 capillaries and a total area of 419723 pixels; and (b) participant 3 after tape-stripping with 484 capillaries and the total area of 54970 pixels. The input image and detection result are shown in the top and bottom rows, respectively.

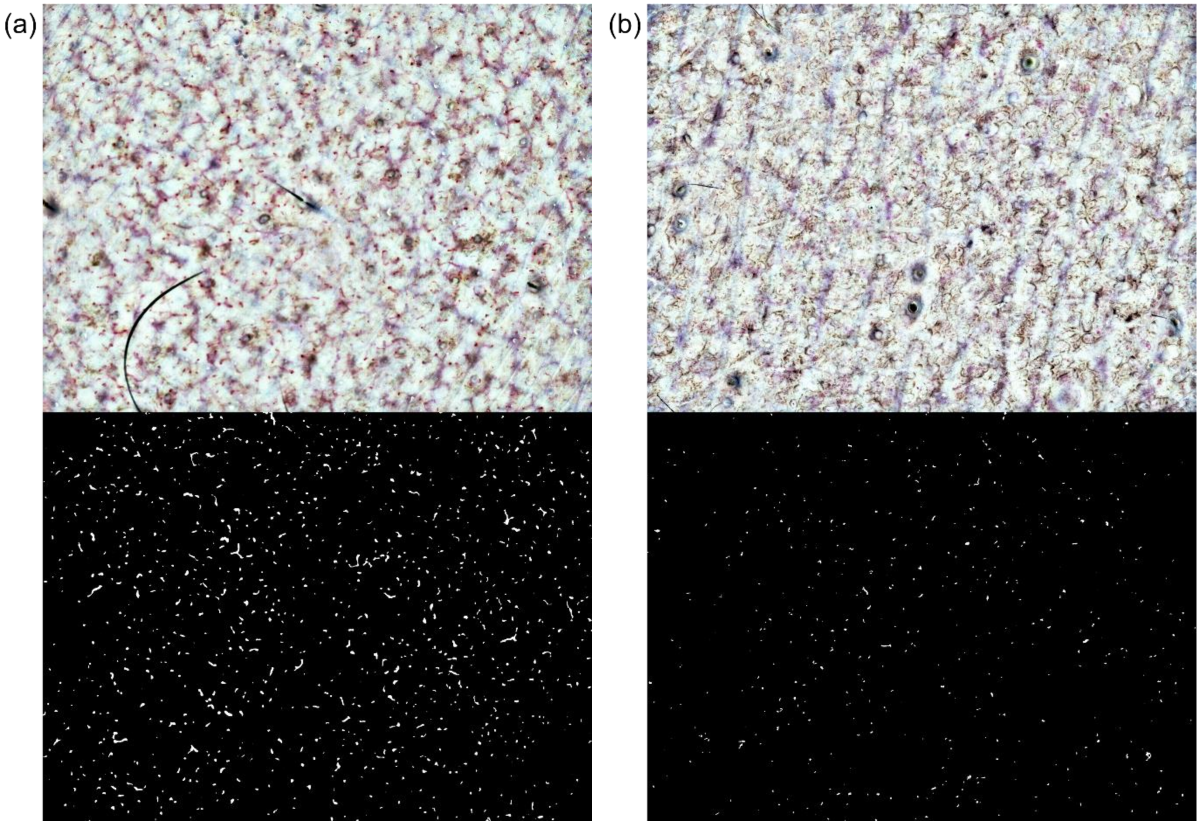

Supplement: Supplementary file 1 [file JBO_028_106003_SD001.pdf]
